# Supplementary material for: Quantitative time-resolved analysis reveals intricate, differential regulation of standard- and immuno-proteasomes
Source: eLife. 2015 Sep 22;4:e07545. doi: 10.7554/eLife.07545 (PMC4611054; doi:10.7554/eLife.07545)
Supplement: Figure 2—source data 2. — The parameters for all models shown in Figure 2 are defined in this table. All of them were unknown and had to be inferred from experimental data. DOI: http://dx.doi.org/10.7554/eLife.07545.011 [file elife07545s002.zip › Figure2_sourceData2.pdf]

Figure 2 - source data 2: Quantitative time-resolved analysis reveals intricate, differential regulation of standard and immuno-proteasomes.

Juliane Liepe, Hermann-Georg Holzhütter, Elena Bellavista, Peter M. Kloetzel, Michael P. H. Stumpf, Michele Mishto

Table 1: List of mathematical model parameters

peptide-bond hydrolysis	
k_p	peptide-bond hydrolysis rate at active site(s)
K_{aS}, K_{aP}	dissociation constant of substrate (S) and product (P) to active site(s)
n_a	Hill coefficient for binding to active site(s)
K_{iS}, K_{iP}	dissociation constant of substrate (S) and product (P) to inhibitor site(s)
n_i	Hill coefficient for binding to inhibitor site(s)
α	factor, by which K_{aS}, K_{aP}, K_{iS} and K_{iP} are multiplied
β	factor, by which k_p is multiplied upon binding to inhibitory site(s)
transport	
k_{on}	association rate to the gate
k_{off}	dissociation rate to from gate
v_{in}	peptide influx rate
τ	peptide translocation rate inside the chamber
v_{out}	peptide efflux rate
C	capacity (maximum number of molecules inside the chamber)
transport regulation	
R_{on}	binding rate to the enhancing regulator site(s)
R_{off}	unbinding rate to the enhancing regulator site(s)
X_{enh}	strength of enhancing regulator site(s)
I_{on}	binding rate to the inhibiting regulator site(s) outside the chamber
I_{off}	unbinding rate to the inhibiting regulator site(s) outside the chamber
h	Hill coefficient for binding to inhibiting regulator site(s) outside the chamber
Y_{inn}	strength of inhibiting regulator site(s)
