# Supplementary material for: Quantitative time-resolved analysis reveals intricate, differential regulation of standard- and immuno-proteasomes
Source: eLife. 2015 Sep 22;4:e07545. doi: 10.7554/eLife.07545 (PMC4611054; doi:10.7554/eLife.07545)
Supplement: Figure 2—source data 3. — The species for all models shown in Figure 2 are defined. The initial conditions are known and defined in Supplementary file 1. DOI: http://dx.doi.org/10.7554/eLife.07545.012 [file elife07545s003.zip › Figure2_sourceData3.pdf]

Figure 2 - source data 3:
Quantitative time-resolved analysis reveals intricate, differential regulation of standard and immuno-proteasomes.

Juliane Liepe, Hermann-Georg Holzhütter, Elena Bellavista, Peter M. Kloetzel,
Michael P. H. Stumpf, Michele Mishto

Table 1: List of mathematical model species

peptide-bond hydrolysis	
E_0	initial proteasome concentration
S	substrate inside the chamber
P	product inside the chamber
transport	
S_{out}	substrate outside the chamber
P_{out}	product outside the chamber
G_1	proteasome gate for influx
G_2	proteasome gate for efflux
$[G_1S_{out}]$	substrate bound to proteasome gate for influx
$[G_1P_{out}]$	product bound to proteasome gate for influx
$[G_2S]$	substrate bound to proteasome gate for efflux
$[G_2P]$	product bound to proteasome gate for efflux
transport regulation	
E_{reg}	enhancing regulator site
$[E_{reg}S_{out}]$	substrate bound to enhancing regulator site outside the chamber
$[E_{reg}P_{out}]$	product bound to enhancing regulator site outside the chamber
$[E_{reg}S]$	substrate bound to enhancing regulator site inside the chamber
$[E_{reg}P]$	product bound to enhancing regulator site inside the chamber
I_0	initial concentration of inhibiting regulator site outside the chamber
I_{free}	inhibiting regulator site outside the chamber
$[IS]$	substrate bound to inhibiting regulator site outside the chamber
$[IP]$	product bound to inhibiting regulator site outside the chamber
