# Supplementary material for: Quantitative time-resolved analysis reveals intricate, differential regulation of standard- and immuno-proteasomes
Source: eLife. 2015 Sep 22;4:e07545. doi: 10.7554/eLife.07545 (PMC4611054; doi:10.7554/eLife.07545)
Supplement: Supplementary file 1. — DOI: http://dx.doi.org/10.7554/eLife.07545.026 [file elife07545s004.pdf]

Supplemental Information: Quantitative time-resolved analysis reveals intricate, differential regulation of standard and immuno-proteasomes.

Juliane Liepe, Hermann-Georg Holzhütter, Elena Bellavista, Peter M. Kloetzel, Michael P. H. Stumpf, Michele Mishto

1 Experimental procedures

1.1 20S proteasome purification and protein homogenates

20S proteasomes are purified from LcL and T2 cells as previously described (Mishto et al., 2010). Lymphoblastoid cell lines (LcLs) are human B lymphocytes immortalized with Epstein Barr virus, which mainly express i-proteasomes (Mishto et al., 2006). T2 cell line is a human T cell leukemia/B cell line hybrid defective in β1i and β5i subunits (Riberdy and Cresswell, 1992). Mouse proteasomes are purified from liver of adult B57CL6 mice as previously described (Mishto et al., 2014). Purity of proteasome preparation has been shown in other studies (Mishto et al., 2012, 2014). Cell protein homogenates are extracted from T2 cells as previously described (Mishto et al., 2006).

1.2 In vitro digestion of synthetic polypeptides and short fluorogenic peptides.

Synthetic polypeptides (40 μM) and short fluorogenic peptides Suc-LLVY-, Bz-VGR- and Z-LLE-MCA are digested by $0.1-2\mu$g purified 20S proteasomes or 5 μg cell protein homogenates (Fig. 1- figure supplement 1B, C) in 100 μl TEAD buffer (Tris 20 mM, EDTA 1 mM, NaN3 1 mM, DTT 1 mM, pH 7.2) over time at 37°C as previously described (Mishto et al., 2006, 2012). In particular, we use: in Fig. 1A-B, Fig. 1 - figure supplement 3 and 5, Fig. 2 - figure supplement 1 and 2, Fig. 3 - figure supplement 1 - 0.12 μg 20S mouse proteasome; in Fig. 1C, Fig. 1 - figure supplement 1A, D, E, 1μg 20S mouse proteasome; in Fig. 1D and Fig. 1 - figure supplement 1D-E, 2 μg 20S mouse proteasome (i.e. 1 and 2 μg 20S mouse proteasome for digesting substrates gp100$_{35-57}$ and LLO$_{291-317}$, respectively); in Fig. 3 and Fig. 1 - figure supplement 4, 0.25 μg 20S mouse proteasome; Fig. 5, Fig. 4 - figure supplement 1A, B, Fig. 5 - fig. supplement 1 0.5 μg T2 and LcL 20S proteasomes; Fig. 4 - fig. supplement 1C and 1D 1 - 2 μg T2 or LcL proteasome (i.e. 1 and 2 μg 20S proteasome for digesting substrates gp100$_{35-57}$ and LLO$_{291-317}$, respectively); All solutions are warmed at 37°C prior the beginning of the reaction. All experiments reported in this study are repeated and measured at least twice.

1.3 Peptide synthesis and quantitation.

The sequence enumeration for the polypeptides gp100$_{35-57}$ (VSRQLRTKAWNRQLYPEWTEAQR) and LLO$_{291-317}$ (AYISSVAYGRQVYLKLSTNSHSTKVKA) are referred to the human protein $g100^{PMEL17}$ and the murine *Listeria monocytogenes's* Listeriolysin O protein (LLO), respectively. Peptides referred as Rpt2 (GTPEGLYL) and Rpt5 (KKKANLQYYA) are the C-terminal sequences of the 19S subunits Rpt2 and Rpt5,

which showed to activate the proteasome hydrolysis by opening the 20S proteasome gate (Gillette et al., 2008). Peptides are synthesized using Fmoc solid phase chemistry as previously described (Mishto et al., 2008). Liquid chromatography mass spectrometry (LC-MS) analyses of polypeptide digestion products are performed as previously described (Liepe et al., 2010b) with the ESI-ion trap instrument DECA XP MAX (ThermoFisher Scientific, USA). Database searching is performed using SpliceMets ProteaJ algorithm (Liepe et al., 2010b). Quantification of produced peptides - both cleavage and spliced products (Liepe et al., 2010b) - and computation of the substrate site-specific cleavage strength (SCS) are carried out by applying QME method to the LC-MS analyses. SCS describes the relative frequencies of proteasome cleavage after any given residue of the synthetic polypeptide substrates (Mishto et al., 2012). To compute the average length of the peptides produced by the proteolysis of the synthetic polypeptides by 20S proteasomes we multiply the absolute amount of each peptide product for its number of residues and compute the average at each time point of the kinetic (Fig. 1 - figure supplement 1D-E and Fig. 4 - figure supplement 2C-D).

2 Mathematical modelling and model inference.

The model development is explained in the results section of the main manuscript. The mathematical models are represented by sets of ordinary differential equations (ODEs) and are shown in the following sections. We extend the ABC-SysBio software (Liepe et al., 2010a, 2014) to be suitable for data sets generated under different initial conditions. ABC-SysBio is applied for model selection and to estimate the model parameters. The applied algorithm in this software is approximate Bayesian computation in a sequential Monte-Carlo framework (ABC-SMC) (Toni et al., 2009). All priors and kernels are uniformly distributed. We chose a population size of 1000 parameter combinations. As distance function we use

$$d = \sum_i \frac{(x_i + x_i\epsilon - x_i^*)^2}{x_i^*}, \tag{1}$$

where x^* are the experimental measurements, x are the simulation results and ϵ is a random variable drawn from a normal distribution with mean 0 and variance 0.1 (error term). In this way the model is fitted to the scaled mean of the experimental data under consideration of the experimental error. Latter is proportionally higher for higher measurement values. For this reason we chose the error term to be multiplicative. The variance is directly computed from the sets of repeated experiments. The ODEs are solved numerically using LSODA algorithm. For efficient simulation a CUDA implementation of LSODA is used from the package cuda-sim (Zhou et al., 2011). In the following sections the species are denoted as defined in Fig. 2 - source data 3, the model parameters are defined in Fig. 2 - source data 2. The ABC-SMC algorithm results in posterior parameter distributions rather than point estimates, which provide us with parameter confidence intervals. All remaining data analysis is performed in *R* (R Core Team, 2014).

2.1 Michaelis-Menten model (MM-model)

This model is represented in Fig. 2A. We use the quasi-steady state assumption for the mathematical implementation.

$$v_{hyd} = \frac{dP}{dt} = \frac{k_p E_0 S}{K_M + S} \tag{2}$$

2.2 Substrate inhibition model (SI-model)

This model is represented in Fig. 2B. This model also makes use of the quasi-steady state assumption and it is an adaptation of the two-site modifier scheme by Schmidtke *et al.* (Schmidtke et al., 2000).

$$v_{hydr} = \frac{n_a k_p E_0 S^{n_a}}{x K_{aS}}\left(1 + \frac{\beta S^{n_i}}{\alpha K_{iS}} + \frac{\beta P^{n_i}}{\alpha K_{iP}}\right) \tag{3}$$

$$x = 1 + \frac{S^{n_a}}{K_{aS}} + \frac{S^{n_i}}{K_{iS}} + \frac{P^{n_a}}{K_{aP}} + \frac{P^{n_i}}{K_{iP}} + \frac{S^{n_a+n_i}}{\alpha K_{aS}K_{iS}} + \frac{P^{n_a+n_i}}{\alpha K_{aP}K_{iP}} + \frac{S^{n_a}P^{n_i}}{\alpha K_{aS}K_{iP}} + \frac{S^{n_i}P^{n_a}}{\alpha K_{iS}K_{aP}} \tag{4}$$

2.3 Dynamic regulator models

These two models are represented in Fig. 2C-D. They are based on the SI-model and extended to allow for a positive feedback loop.

$$v_{hydr} = \frac{n_a R_2 E_0 S^{n_a}}{x K_{aS}}\left(1 + \frac{\beta S^{n_i}}{\alpha K_{iS}} + \frac{\beta P^{n_i}}{\alpha K_{iP}}\right) \tag{5}$$

$$x = 1 + \frac{S^{n_a}}{R_1 K_{aS}} + \frac{S^{n_i}}{K_{iS}} + \frac{P^{n_a}}{R_1 K_{aP}} + \frac{P^{n_i}}{K_{iP}} + \frac{S^{n_a+n_i}}{\alpha R_1 K_{aS}K_{iS}} + \frac{P^{n_a+n_i}}{\alpha R_1 K_{aP}K_{iP}} + \frac{S^{n_a}P^{n_i}}{\alpha R_1 K_{aS}K_{iP}} + \frac{S^{n_i}P^{n_a}}{\alpha K_{iS}R_1 K_{aP}}, \tag{6}$$

with $R_1 = \frac{1}{(1+PX_{enh})}$ and $R_2 = k_p$ for model in Fig. 3C and $R_1 = 1$ and $R_2 = k_p(1 + PX_{enh})$ for model in Fig. 3D

2.4 Compartment Model

The following models are represented in Fig. 2E-J. These models have the same mathematical description for the catalytic events inside the proteasome chamber (allosteric substrate and product inhibition, see SI-model), described as follows. They differ in the mechanism of peptide transport and transport regulation.

S and P denote the substrate and product concentration per proteasome chamber.

$$v_{hydr} = \frac{n_a k_p E_0 S^{n_a}}{x K_{aS}}\left(1 + \frac{\beta S^{n_i}}{\alpha K_{iS}} + \frac{\beta P^{n_i}}{\alpha K_{iP}}\right) \tag{7}$$

$$x = 1 + \frac{S^{n_a}}{K_{aS}} + \frac{S^{n_i}}{K_{iS}} + \frac{P^{n_a}}{K_{aP}} + \frac{P^{n_i}}{K_{iP}} + \frac{S^{n_a+n_i}}{\alpha K_{aS}K_{iS}} + \frac{P^{n_a+n_i}}{\alpha K_{aP}K_{iP}} + \frac{S^{n_a}P^{n_i}}{\alpha K_{aS}K_{iP}} + \frac{S^{n_i}P^{n_a}}{\alpha K_{iS}K_{aP}} \tag{8}$$

2.4.1 Transport with association to the gate - no regulation

This model is represented in Fig. 2F. S and P denote the total substrate and product concentration in all proteasome chambers.

$$\frac{dS_{out}}{dt} = -S_{out}G_1k_{on} + [G_1S_{out}]k_{off} + [G_2S]v_{out} \tag{9}$$

$$\frac{dG_1}{dt} = -(S_{out} + P_{out})G_1k_{on} + ([G_1S_{out}] + [G_1P_{out}])(k_{off} + transport_{in}) \tag{10}$$

$$\frac{d[G_1S_{out}]}{dt} = S_{out}G_1k_{on} - [G_1S_{out}](k_{off} + transport_{in}) \tag{11}$$

$$\frac{dP_{out}}{dt} = -P_{out}G_1k_{on} - [G_1P_{out}]k_{off} + [G_2P]v_{out} \tag{12}$$

$$\frac{d[G_1P_{out}]}{dt} = P_{out}G_1k_{on} - [G_1P_{out}](k_{off} + transport_{in}) \tag{13}$$

$$\frac{dS_{in}}{dt} = [G_1S_{out}]transport_{in} - \tau\frac{SG_2}{E_0} - v_{hydr} \tag{14}$$

$$\frac{dP_{in}}{dt} = [G_1P_{out}]transport_{in} - \tau\frac{PG_2}{E_0} + v_{hydr} \tag{15}$$

$$\frac{dG_2}{dt} = -\tau\frac{G_2(S+P)}{E_0} + ([G_2S] + [G_2P])v_{out} \tag{16}$$

$$\frac{d[G_2S]}{dt} = \tau\frac{G_2S}{E_0} - [G_2S]v_{out} \tag{17}$$

$$\frac{d[G_2P]}{dt} = \tau\frac{G_2P}{E_0} - [G_2P]v_{out} \tag{18}$$

$$transport_{in} = v_{in}tanh(E_0C - S - P) \tag{19}$$

2.4.2 Transport with association to the gate - enhancer site outside the proteasome chamber

This model is represented in Fig. 2G and I. If $I_{on} = 0$ the model contains no inhibitory site (Fig. 2G). If $I_{on} > 0$ the model contains the inhibitory site outside the chamber (Fig. 2I). The enhancing regulatory site affects the terms $transport_{in}$ and $transport_{out}$. The inhibitory regulatory site outside the chamber can reduce the gate opening.

$$\begin{aligned}\frac{dS_{out}}{dt} =& - S_{out}G_1k_{on} + [G_1S_{out}]k_{off} + [G_2S]transport_{out} - h{S_{out}}^hI_{free}I_{on} + h[IS]I_{off} \\ & - R_{on}S_{out}E_{reg} + R_{off}[E_{reg}S_{out}]\end{aligned} \tag{20}$$

$$\frac{dG_1}{dt} = -(S_{out} + P_{out})G_1k_{on} + ([G_1S_{out}] + [G_1P_{out}])(k_{off} + transport_{in}) \tag{21}$$

$$\frac{d[G_1S_{out}]}{dt} = S_{out}G_1k_{on} - [G_1S_{out}](k_{off} + transport_{in}) \tag{22}$$

$$\begin{aligned}\frac{dP_{out}}{dt} =& - P_{out}G_1k_{on} - [G_1P_{out}]k_{off} + [G_2P]transport_{out} - h{P_{out}}^hI_{free}I_{on} + h[IP]I_{off} \\ & - R_{on}P_{out}E_{reg} + R_{off}[E_{reg}P_{out}]\end{aligned} \tag{23}$$

$$\frac{d[G_1P_{out}]}{dt} = P_{out}G_1k_{on} - [G_1P_{out}](k_{off} + transport_{in}) \tag{24}$$

$$\frac{dS}{dt} = [G_1S_{out}]transport_{in} - \tau\frac{SG_2}{E_0} - v_{hydr} \tag{25}$$

$$\frac{dP}{dt} = [G_1P_{out}]transport_{in} - \tau\frac{PG_2}{E_0} + v_{hydr} \tag{26}$$

$$\frac{dG_2}{dt} = -\tau\frac{G_2(S+P)}{E_0} + ([G_2S] + [G_2P])transport_{out} \tag{27}$$

$$\frac{d[G_2S]}{dt} = \tau\frac{G_2S}{E_0} - [G_2S]transport_{out} \tag{28}$$

$$\frac{d[G_2P]}{dt} = \tau\frac{G_2P}{E_0} - [G_2P]transport_{out} \tag{29}$$

$$\frac{dE_{reg}}{dt} = -R_{on}E_{reg}(S_{out} + P_{out}) + R_{off}([E_{reg}S_{out}] + [E_{reg}P_{out}]) \tag{30}$$

$$\frac{d[E_{reg}S_{out}]}{dt} = R_{on}E_{reg}S_{out} - R_{off}[E_{reg}S_{out}] \tag{31}$$

$$\frac{d[E_{reg}P_{out}]}{dt} = R_{on}E_{reg}P_{out} - R_{off}[E_{reg}P_{out}] \tag{32}$$

$$\frac{dI_{free}}{dt} = -({S_{out}}^h + {P_{out}}^h)I_{free}I_{on} + I_{off}([IS] + [IP]) \tag{33}$$

$$\frac{d[IS]}{dt} = {S_{out}}^hI_{free}I_{on} - I_{off}[IS] \tag{34}$$

$$\frac{d[IP]}{dt} = {P_{out}}^hI_{free}I_{on} - I_{off}[IP] \tag{35}$$

$$transport_{in} = v_{in}\left(1 + \frac{X_{enh}\frac{[E_{reg}S]+[E_{reg}P]}{2E_0}}{1 + Y_{inh}\frac{[IS]+[IP]}{I_0}}\right)tanh(E_0C - S - P) \tag{36}$$

$$transport_{out} = v_{out}\left(1 + \frac{X_{enh}\frac{[E_{reg}S]+[E_{reg}P]}{2E_0}}{1 + Y_{inh}\frac{[IS]+[IP]}{I_0}}\right) \tag{37}$$

2.4.3 Transport with association to the gate - enhancer site inside the proteasome chamber

This model is represented in Fig. 2H and J. If $I_{on} = 0$ the model contains no inhibitory site (Fig.2 H). If $I_{on} > 0$ the model contains the inhibitory site outside the chamber (Fig.2J). All other model assumptions are as above.

$$\frac{dS_{out}}{dt} = -S_{out}G_1k_{on} + [G_1S_{out}]k_{off} + [G_2S]transport_{out} - h{S_{out}}^hI_{free}I_{on} + h[IS]I_{off} \tag{38}$$

$$\frac{dG_1}{dt} = -(S_{out} + P_{out})G_1k_{on} + ([G_1S_{out}] + [G_1P_{out}])(k_{off} + transport_{in}) \tag{39}$$

$$\frac{d[G_1S_{out}]}{dt} = S_{out}G_1k_{on} - [G_1S_{out}](k_{off} + transport_{in}) \tag{40}$$

$$\frac{dP_{out}}{dt} = -P_{out}G_1k_{on} - [G_1P_{out}]k_{off} + [G_2P]transport_{out} - h{P_{out}}^hI_{free}I_{on} + h[IP]I_{off} \tag{41}$$

$$\frac{d[G_1P_{out}]}{dt} = P_{out}G_1k_{on} - [G_1P_{out}](k_{off} + transport_{in}) \tag{42}$$

$$\frac{dS}{dt} = [G_1 S_{out}]transport_{in} - \tau \frac{SG_2}{E_0} - v_{hydr} - R_{on}\frac{SE_{reg}}{E_0} + R_{off}[E_{reg}S] \tag{43}$$

$$\frac{dP}{dt} = [G_1 P_{out}]transport_{in} - \tau \frac{PG_2}{E_0} + v_{hydr} - R_{on}\frac{PE_{reg}}{E_0} + R_{off}[E_{reg}P] \tag{44}$$

$$\frac{dG_2}{dt} = -\tau \frac{G_2(S+P)}{E_0} + ([G_2S] + [G_2P])transport_{out} \tag{45}$$

$$\frac{d[G_2S]}{dt} = \tau \frac{G_2S}{E_0} - [G_2S]transport_{out} \tag{46}$$

$$\frac{d[G_2P]}{dt} = \tau \frac{G_2P}{E_0} - [G_2P]transport_{out} \tag{47}$$

$$\frac{dE_{reg}}{dt} = -\frac{R_{on}E_{reg}}{E_0}(S+P) + R_{off}([E_{reg}S] + [E_{reg}P]) \tag{48}$$

$$\frac{d[E_{reg}S]}{dt} = \frac{R_{on}E_{reg}}{E_0}S - R_{off}[E_{reg}S] \tag{49}$$

$$\frac{d[E_{reg}P]}{dt} = \frac{R_{on}E_{reg}}{E_0}P - R_{off}[E_{reg}P] \tag{50}$$

$$\frac{dI_{free}}{dt} = -({S_{out}}^h + {P_{out}}^h)I_{free}I_{on} + I_{off}([IS] + [IP]) \tag{51}$$

$$\frac{d[IS]}{dt} = {S_{out}}^h I_{free}I_{on} - I_{off}[IS] \tag{52}$$

$$\frac{d[IP]}{dt} = {P_{out}}^h I_{free}I_{on} - I_{off}[IP] \tag{53}$$

$$transport_{in} = v_{in}\left(1 + \frac{X_{enh}\frac{[E_{reg}S]+[E_{reg}P]}{2E_0}}{1 + Y_{inh}\frac{[IS]+[IP]}{I_0}}\right)tanh(E_0C - S - P) \tag{54}$$

$$transport_{out} = v_{out}\left(1 + \frac{X_{enh}\frac{[E_{reg}S]+[E_{reg}P]}{2E_0}}{1 + Y_{inh}\frac{[IS]+[IP]}{I_0}}\right) \tag{55}$$

2.4.4 Free transport of substrate and product

This model is represented in Fig. 2E.

$$\frac{dS_{out}}{dt} = -S_{out}transport_{in} + transport_{out}S \tag{56}$$

$$\frac{dP_{out}}{dt} = -P_{out}transport_{in} + transport_{out}P \tag{57}$$

$$\frac{dS}{dt} = S_{out}transport_{in} - transport_{out}S - v_{hydr} \tag{58}$$

$$\frac{dP}{dt} = P_{out}transport_{in} - transport_{out}P + v_{hydr} \tag{59}$$

$$transport_{in} = v_{in}tanh(E_0C - S - P) \tag{60}$$

$$transport_{out} = v_{out} \tag{61}$$

2.5 Initial conditions

The initial conditions of the following species are 0nM in all models: $[G_1S_{out}]$, $[G_1P_{out}]$, $[G_2S]$, $[G_2P]$, P, P_{out}, $[E_{reg}S]$, $[E_{reg}P]$, $[IS]$ and $[IP]$. E_0 is 1.66nM and 6.66nM when using mouse proteasome and human (s- and i-) proteasome, respectively. The initial conditions of G_1, G_2 and E_{reg} are $2E_0$. The initial condition for I_{free} is E_0 (equivalent to I_0). The initial conditions for S_{out} depend on the substrates and proteasome used. For mouse proteasome using Suc-LLVY-MCA S_{out} is 10μM, 20μM, 40μM, 80μM, 160μM, 240μM and 480μM for each data set, respectively. For mouse proteasome using Bz-VGR-MCA and Z-LLE-MCA S_{out} is 20μM, 40μM, 80μM, 160μM, 240μM, 480μM and 640μM for each data set, respectively. For human s- and i-proteasome the initial conditions for S_{out} are the same for all three substrates: 20μM, 40μM, 80μM, 160μM, 320μM and 640μM for each data set, respectively. The initial conditions of S are equivalent to those of S_{out} for non-compartmentalised models; 0nM for compartmentalised models.

2.6 In silico predictions

All *in silico* simulations are based on sampled parameters from the posterior parameter distribution. The open gate mutant is simulated by increasing the rates for v_{in} and v_{out} 20-fold, while no more positive or negative regulation is possible ($X_{enh} = Y_{inh} = 0$). Similarly we performed the *in silico* prediction with Rpt peptide. Here we increased the rates for v_{in} and v_{out} by a factor, which is calibrated for each substrate (Suc-LLVY-MCA: 5; Bz-VGR-MCA: 20; and Z-LLE-MCA: 15). The *in silico* predictions in present of the peptide LLVY are based on the posterior parameter distributions of the relevant substrate. We extend the model by adding a further species (LLVY peptide, initial condition: 50μM) to the system. Furthermore, species describing LLVY bound to receptors are added. The parameters for the reactions involving LLVY peptide are sampled from the posterior parameter distribution inferred from Suc-LLVY-MCA degradation kinetics. We assume no competing effects of the substrate and LLVY peptide at the gate, but for all other binding sites. For all *in silico* experiments we sample 500 parameter combinations from the posterior parameter distributions and simulate each set. Shown in this work are the means of all 500 simulations.

2.7 Analysis of rate limiting steps

To determine the rate limiting step of the substrate hydrolysis we need to determine the reaction to which the product formation is most sensitive. Additionally this reaction needs to have the ability not only to change the product formation, but to increase it. We simulate the model with 100 parameter combinations sampled from the corresponding posterior parameter distribution and increase a chosen reaction by a factor between 1 and 10. The resulting product formation after 60 min is then compared to the unchanged model output and the fold change in product is computed. We finally plot the mean fold change of the 100 simulations. We change the following reactions: affinity to the gate, peptide influx, hydrolysis, peptide transport inside the chamber, peptide efflux and the gate size, which controls peptide influx and efflux.

References

Barnes, C. P., Silk, D., Sheng, X., and Stumpf, M. P. (2011). Bayesian design of synthetic biological systems. *Proceedings of the National Academy of Sciences*, 108(37):15190–15195.

Gillette, T. G., Kumar, B., Thompson, D., Slaughter, C. A., and DeMartino, G. N. (2008). Differential roles of the cooh termini of aaa subunits of pa700 (19 s regulator) in asymmetric assembly and activation of the 26 s proteasome. *Journal of Biological Chemistry*, 283(46):31813–31822.

Liepe, J., Barnes, C., Cule, E., Erguler, K., Kirk, P., Toni, T., and Stumpf, M. P. (2010a). Abc-sysbio—approximate bayesian computation in python with gpu support. *Bioinformatics*, 26(14):1797–1799.

Liepe, J., Kirk, P., Filippi, S., Toni, T., Barnes, C. P., and Stumpf, M. P. (2014). A framework for parameter estimation and model selection from experimental data in systems biology using approximate bayesian computation. *Nature protocols*, 9(2):439–456.

Liepe, J., Mishto, M., Textoris-Taube, K., Janek, K., Keller, C., Henklein, P., Kloetzel, P. M., and Zaikin, A. (2010b). The 20s proteasome splicing activity discovered by splicemet. *PLoS computational biology*, 6(6):e1000830.

Mishto, M., Bellavista, E., Ligorio, C., Textoris-Taube, K., Santoro, A., Giordano, M., D'Alfonso, S., Listì, F., Nacmias, B., Cellini, E., et al. (2010). Immunoproteasome lmp2 60hh variant alters mbp epitope generation and reduces the risk to develop multiple sclerosis in italian female population. *PloS one*, 5(2):e9287.

Mishto, M., Goede, A., Taube, K. T., Keller, C., Janek, K., Henklein, P., Niewienda, A., Kloss, A., Gohlke, S., Dahlmann, B., et al. (2012). Driving forces of proteasome-catalyzed peptide splicing in yeast and humans. *Molecular & Cellular Proteomics*, 11(10):1008–1023.

Mishto, M., Liepe, J., Textoris-Taube, K., Keller, C., Henklein, P., Weberruß, M., Dahlmann, B., Enenkel, C., Voigt, A., Kuckelkorn, U., et al. (2014). Proteasome isoforms exhibit only quantitative differences in cleavage and epitope generation. *European journal of immunology*, 10.1002/eji.201444902.

Mishto, M., Luciani, F., Holzhütter, H.-G., Bellavista, E., Santoro, A., Textoris-Taube, K., Franceschi, C., Kloetzel, P. M., and Zaikin, A. (2008). Modeling the¡ i¿ in vitro¡/i¿ 20s proteasome activity: The effect of pa28–$\alpha\beta$ and of the sequence and length of polypeptides on the degradation kinetics. *Journal of molecular biology*, 377(5):1607–1617.

Mishto, M., Santoro, A., Bellavista, E., Sessions, R., Textoris-Taube, K., Piaz, F. D., Carrard, G., Forti, K., Salvioli, S., Friguet, B., et al. (2006). A structural model of 20s immunoproteasomes: effect of lmp2 codon 60 polymorphism on expression, activity, intracellular localisation and insight into the regulatory mechanisms. *Biological chemistry*, 387(4):417–429.

R Core Team (2014). *R: A Language and Environment for Statistical Computing*. R Foundation for Statistical Computing, Vienna, Austria.

Riberdy, J. and Cresswell, P. (1992). The antigen-processing mutant t2 suggests a role for mhc-linked genes in class ii antigen presentation. *The Journal of Immunology*, 148(8):2586–2590.

Schmidtke, G., Emch, S., Groettrup, M., and Holzhütter, H.-G. (2000). Evidence for the existence of a non-catalytic modifier site of peptide hydrolysis by the 20 s proteasome. *Journal of Biological Chemistry*, 275(29):22056–22063.

Toni, T., Welch, D., Strelkowa, N., Ipsen, A., and Stumpf, M. P. (2009). Approximate bayesian computation scheme for parameter inference and model selection in dynamical systems. *Journal of the Royal Society Interface*, 6(31):187–202.

Zhou, Y., Liepe, J., Sheng, X., Stumpf, M. P., and Barnes, C. (2011). Gpu accelerated biochemical network simulation. *Bioinformatics*, 27(6):874–876.
